# Intervenção de esportes modificados para melhorar metas de participação e competências de atividade em crianças deambuladoras com paralisia cerebral: um ensaio clínico randomizado

**DOI:** 10.1111/dmcn.16411

**Published:** 2025-07-03

**Authors:** Ricardo R. Sousa Junior, Georgina L. Clutterbuck, Rafaela F. Guimaraes, Mariane G. Souza, Luana C. Silva, F. Virginia Wright, Ana Cristina R. Camargos, Hércules R. Leite

**Affiliations:** ^1^ Programa de Pós‐Graduação em Ciências da Reabilitação, Escola de Educação Física, Fisioterapia e Terapia Ocupacional Universidade Federal de Minas Gerais MG Brasil; ^2^ Escola de Ciências da Saúde e Reabilitação Universidade de Queensland Brisbane QLD Austrália; ^3^ Hospital de Reabilitação Infantil Holland Bloorview Toronto ON Canadá; ^4^ Departamento de Fisioterapia Universidade de Toronto Toronto ON Canadá; ^5^ Centro CanChild de Pesquisa sobre Deficiência na Infância Universidade McMaster Hamilton ON Canadá

## Abstract

**Objetivo:**

Avaliar a eficácia de uma intervenção de esportes modificados (Sports Stars Brasil) nos objetivos de participação em atividades físicas de lazer, desempenho e capacidade motora, funções corporais, níveis de atividade física, alfabetização física e participação geral em crianças deambuladoras com paralisia cerebral (PC).

**Método:**

Neste ensaio clínico randomizado, 38 crianças deambuladoras com PC (21 meninos, 17 meninas; idades de 6 a 12 anos) foram designadas aleatoriamente para o grupo de intervenção ou controle. O grupo de intervenção participou de oito sessões semanais de 1 hora de esportes modificados, com foco em treinamento motor em grupo e introdução a esportes populares brasileiros. O grupo controle recebeu a fisioterapia habitual. Os desfechos foram avaliados no início, ao final da intervenção e após 12 semanas. Modelos lineares mistos foram utilizados para a análise.

**Resultados:**

O programa Sports Stars Brasil foi mais eficaz do que o cuidado habitual na melhoria da participação em atividades físicas de lazer e no desempenho motor, tanto imediatamente após a intervenção quanto no acompanhamento. Os grupos apresentaram resultados similares em capacidade motora, alfabetização física, funções corporais, níveis de atividade física e participação geral logo após a intervenção. No acompanhamento de 12 semanas, o grupo Sports Stars apresentou maiores melhorias na capacidade motora e na atividade física de moderada a vigorosa intensidade.

**Interpretação:**

O Sports Stars Brasil é uma intervenção promissora e de baixo custo para promover a participação e as habilidades motoras em crianças com PC, especialmente em contextos de poucos recursos.

Intervenções de esportes modificados para crianças com Paralisia Cerebral: Resumo gráfico.

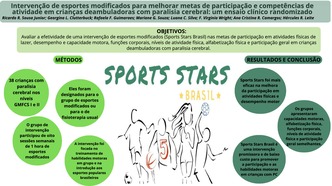

**Abreviações**
COPM: Canadian Occupational Performance Measure
TGMD‐2: Test of Gross Motor Development, Second Edition

**O que este artigo acrescenta**

- Os esportes modificados melhoraram a participação em atividades físicas de lazer em crianças deambuladoras com paralisia cerebral.
- Intervenções de esportes modificados aprimoraram o desempenho motor em habilidades motoras fundamentais.
- Os resultados na capacidade de habilidades motoras foram comparáveis aos da fisioterapia habitual.
- Foram encontrados resultados semelhantes em funções corporais, alfabetização física e participação geral.
- Os níveis de atividade física e a capacidade motora melhoraram no follow‐up no grupo de intervenção.

Crianças deambuladoras com paralisia cerebral (PC) que são classificadas nos níveis I e II do Sistema de Classificação da Função Motora Grossa (GMFCS)^1^ podem andar sem dispositivos de assistência na maioria dos ambientes, mas têm dificuldades com habilidades motoras como correr, pular e arremessar.^1^ Essas limitações de atividade, juntamente com barreiras pessoais e ambientais, podem levar a menor participação em atividades físicas de lazer (por exemplo, esportes ou recreação física), o que pode resultar em comportamentos sedentários e desfechos de saúde ruins ao longo da vida.^2^, ^3^ Intervenções de esportes modificados podem ajudar essas crianças desenvolvendo habilidades motoras e promovendo a participação futura em esportes ou recreação física.^4^, ^5^, ^6^

As intervenções de esportes modificados estão fundamentadas no Modelo de Participação SPORTS, que delineia uma série de etapas pelas quais crianças e adolescentes com deficiências podem passar enquanto se preparam para, ingressam, participam e se destacam em esportes e recreação física.^7^ As intervenções de esportes modificados representam o ‘P' no acrônimo SPORTS (‘Profissionais liderando grupos de esporte’).^7^ Os esportes modificados conectam intervenções relacionadas à saúde e ao esporte com a atividade física de lazer convencional na comunidade, nas etapas ORTS do Modelo SPORTS de Participação.^7^, ^8^

Nos últimos anos, nosso grupo de pesquisa tem focado em descrever os componentes do tratamento e compreender a efetividade das intervenções de esportes modificados para crianças com deficiências neuromotoras como a PC.^7^, ^8^, ^9^, ^10^, ^11^, ^12^, ^13^, ^14^ Essas intervenções normalmente consistem em dois ingredientes ativos principais: (1) treinamento repetido de habilidades motoras e (2) introdução aos esportes, atuando por meio do mecanismo de ação “aprender fazendo”.^9^ Os principais alvos são a capacidade e o desempenho das habilidades motoras; entretanto, funções corporais (por exemplo, força muscular, equilíbrio e força) e a participação em atividades físicas de lazer são desfechos indiretos que também podem ser alcançados.^9^ Como outras intervenções focadas em esportes, as intervenções de esportes modificados também incluem a conquista de fatores contextuais, como as competências de alfabetização física (por exemplo, componentes físicos, psicológicos, sociais e cognitivos^15^) para efetivamente alcançar mudanças permanentes na participação em atividade física.^8^ Mesmo que os ingredientes do componente físico sejam os mais relatados e claros nas intervenções de esportes modificados, é importante integrar competências sociais, cognitivas e psicológicas relacionadas aos ingredientes, para promover diferenças na participação em atividades físicas de lazer.^8^, ^9^

Um exemplo de intervenção de esportes modificados que integra todos os componentes da alfabetização física é um programa conduzido por profissionais em grupo chamado Sports Stars.^10^ Seu objetivo principal é promover a participação em atividade física focando na alfabetização física em crianças com deficiências. O programa enfatiza o treinamento de habilidades motoras, o desenvolvimento da autoconfiança, o trabalho em equipe e a educação esportiva. Foi originalmente criado na Austrália^10^ e adaptado para o contexto brasileiro (chamado Sports Stars Brasil).^12^ A versão australiana foi bem aceita por pais e fisioterapeutas e eficaz na melhoria da participação em atividades físicas de lazer e habilidades motoras.^10^, ^13^ Pais e cuidadores no Brasil envolvidos em sua primeira implementação indicaram que a versão adaptada é uma intervenção viável para crianças deambuladoras com PC que influenciou positivamente diferentes aspectos de todas as “Minhas palavras favoritas” para o desenvolvimento infantil (funcionalidade, amigos, diversão, família, saúde e futuro).^12^

Embora as intervenções de esportes modificados provavelmente melhorem a participação em atividades físicas de lazer, habilidades motoras e funções corporais em crianças com PC, a qualidade das evidências sobre sua efetividade ainda é baixa devido à falta de estudos metodologicamente robustos.^9^, ^16^ Ensaios clínicos randomizados são necessários para fortalecer a evidência sobre desfechos já investigados, como ganhos em habilidades motoras e participação em atividade física. Além disso, como a participação é fortemente influenciada pelo ambiente das crianças^17^, é crucial avaliar a efetividade das intervenções em países de baixa e média renda, como o Brasil. Sabe‐se que os estudos brasileiros de fisioterapia em PC focados em participação são escassos.^18^ Essa lacuna também afeta a prática clínica, pois muitos fisioterapeutas brasileiros não oferecem intervenções focadas na participação, priorizando funções e estruturas corporais.^19^

Segundo o relatório anual do Registro Brasileiro de Paralisia Cerebral, 89,8% das famílias de pessoas com PC utilizam o sistema público de saúde brasileiro e 23% não praticam nenhum tipo de atividade física.^20^ Longas listas de espera e falta de serviços especializados em fisioterapia são barreiras para indivíduos com PC. Além disso, a assistência à saúde para pessoas com PC no Brasil impõe altos custos ao Sistema Único de Saúde.^21^ Intervenções conduzidas por profissionais em grupo, como o Sports Stars Brasil, podem ser uma alternativa promissora para promover a participação em atividades físicas de lazer e outros importantes desfechos funcionais em crianças com PC.

Um dos principais objetivos do Sports Stars Brasil foi implementar uma intervenção centrada nos esportes visando aumentar a participação em atividades físicas de lazer entre crianças brasileiras com deficiência, enquanto contribui para a pesquisa em esportes modificados, especialmente no contexto de países de baixa e média renda.^12^ Investigações adicionais utilizando o desenho de ensaio clínico randomizado ajudariam a determinar a extensão da efetividade para desfechos ainda não explorados, como níveis de atividade física (incluindo comportamento sedentário e atividade física diária), bem como os domínios social, cognitivo e psicológico da alfabetização física. Além disso, forneceriam insights sobre a participação geral em diferentes contextos de vida, todos essenciais para apoiar o engajamento vitalício em atividade física.

O objetivo principal deste estudo foi avaliar a efetividade de uma intervenção de esportes modificados (Sports Stars Brasil) para crianças deambuladoras com PC, em comparação com a fisioterapia usual, em termos de frequência (ou seja, assiduidade) e envolvimento em atividades físicas de lazer (avaliados por metas selecionadas pelos pais). Os desfechos secundários incluíram desempenho e capacidade de habilidades motoras, níveis de atividade física, domínios da alfabetização física, funções corporais e participação geral.

## MÉTODOS

### Desenho do estudo

Este estudo foi um ensaio clínico randomizado controlado, simples‐cego, com dois grupos (Sports Stars Brasil versus grupo controle de fisioterapia usual), com alocação oculta, avaliação cegada e análise por intenção de tratar. O estudo foi registrado prospectivamente no Registro Brasileiro de Ensaios Clínicos (RBR‐3RWTYW, Número Universal de Ensaios da Organização Mundial da Saúde U1111‐1256‐4998) e aprovado pelo comitê de ética da Universidade Federal de Minas Gerais, Brasil (CAEE 33238520.5.0000.5149). O consentimento informado por escrito foi obtido de todos os pais e crianças antes do início dos procedimentos do estudo. O protocolo do estudo, descrevendo os métodos deste estudo com detalhes, pode ser encontrado em outra publicação.^22^ Este estudo foi conduzido entre novembro de 2021 e março de 2024.

### Local

Este estudo foi realizado nas quadras poliesportivas e pistas de corrida da Escola de Educação Física, Fisioterapia e Terapia Ocupacional da Universidade Federal de Minas Gerais, Minas Gerais, Brasil.

### Amostra do estudo, recrutamento e cálculo amostral

Os participantes foram recrutados por conveniência em instituições públicas ou filantrópicas de fisioterapia e clínicas privadas em Belo Horizonte, Brasil, e regiões próximas. As crianças eram elegíveis para o estudo se tivessem entre 6 e 12 anos de idade no início da intervenção, com diagnóstico de paralisia cerebral (PC) e classificadas nos níveis I ou II do GMFCS. Elas precisavam ser capazes de entender instruções simples e não possuir qualquer condição ortopédica ou cardiorrespiratória que impedisse a participação segura em atividades físicas com colegas. Como as crianças randomizadas para o grupo Sports Stars Brasil seriam temporariamente suspensas do tratamento fisioterapêutico usual (1–2 horas de terapia individual por semana), os pais e terapeutas precisavam inicialmente concordar que a criança poderia se beneficiar do programa e não ter outros objetivos terapêuticos não relacionados ao estudo (por exemplo, função dos membros superiores, alinhamento) que tornassem prejudicial a suspensão da fisioterapia regular. Crianças que não puderam suspender o tratamento fisioterapêutico não foram consideradas elegíveis para inscrição no estudo. O tamanho amostral alvo de 38 crianças foi baseado no estudo original do Sports Stars,^13^ onde foi encontrado um tamanho de efeito de 1,05 na diferença de médias do desfecho de participação (ou seja, metas de frequência e/ou comparecimento definidas pelos pais, medidas por uma administração modificada do Canadian Occupational Performance Measure, COPM)^23^ em comparação com o cuidado usual, após o tratamento.10 O software GPower 3.1.9 (Heinrich Heine University, Düsseldorf, Alemanha) foi utilizado para o cálculo do tamanho da amostra, considerando poder de 80%, α = 5% e 20% de perda na amostra.

### Randomização e cegamento

As crianças foram recrutadas e inscritas pelo principal interventor (RRSJ). As crianças foram randomizadas para o grupo Sports Stars Brasil ou para o grupo controle de fisioterapia usual por um dos investigadores (HRL). Um gerador de números aleatórios criou uma sequência aleatória de números que foram ocultados em envelopes opacos numerados individualmente e lacrados. Essa sequência randomizou as crianças para o grupo Sports Stars Brasil (números pares ocultos) ou para o grupo controle de fisioterapia usual (números ímpares ocultos). A randomização ocorreu em etapas; cada etapa era concluída quando um subgrupo de 8 a 16 participantes era recrutado (para completar um grupo de quatro a oito crianças no grupo de intervenção). Uma nova sequência foi gerada para cada randomização de subgrupo até que 38 crianças fossem alocadas. Devido ao pequeno número de participantes disponíveis e dispostos a participar do estudo, a randomização foi baseada apenas no gerador de números aleatórios e não nas características dos participantes (por exemplo, nível do GMFCS). Os avaliadores estavam cegos quanto à alocação dos grupos.

### Grupos de intervenção

#### Intervenção Sports Stars Brasil

Esta intervenção de esportes modificados, conduzida por profissionais e em grupos, contou com quatro a oito participantes em cada grupo do programa. Foi liderada por um Fisioterapeuta treinado, juntamente com profissionais de Educação Física e aproximadamente quatro a seis estudantes de graduação de ambas as áreas. As crianças no Sports Stars Brasil participaram de oito sessões semanais de 1 hora, com o treinamento focado em quatro esportes diferentes (2 semanas para cada um): futebol, handebol, basquete e atletismo. Intervenções de esportes modificados apresentam parâmetros variados de dosagem;^9^ para este estudo, optou‐se por usar a mesma dosagem e períodos de follow‐up que Clutterbuck et al. (2020) utilizaram no estudo Sports Stars Austrália, porque apresentaram resultados positivos na participação das crianças, além de permitir uma discussão comparativa entre as duas versões do Sports Stars.^13^ Essa baixa dosagem também pode ser adequada para aplicação em países de baixa e média renda, como o Brasil.

Os principais ingredientes ativos dessa intervenção foram a prática ativa repetitiva de habilidades motoras relacionadas aos esportes e a introdução aos esportes‐alvo (futebol, handebol, basquete e atletismo), com modificação da complexidade (ou seja, alteração das regras, número de participantes ou equipamentos para introduzir o esporte). O treinamento das habilidades motoras foi conduzido proporcionando oportunidades de estrutura e prática repetida, utilizando progressão graduada da complexidade das tarefas, dicas verbais, estratégias de feedback e estratégias motivacionais para desenvolver os componentes cognitivos, sociais e psicológicos da alfabetização física.

**FIGURA 1:**
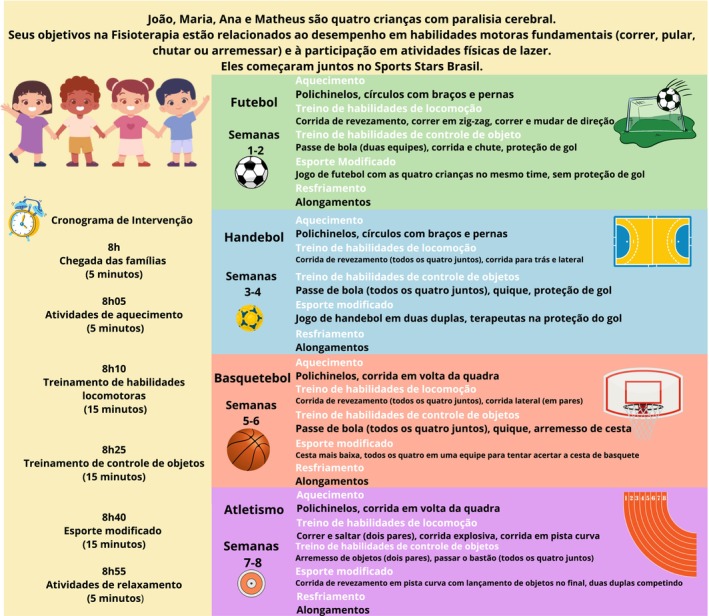
Plano de sessões do Sports Stars Brasil.

O principal interventor (RRSJ) foi um fisioterapeuta pediátrico com 9 anos de experiência com crianças com paralisia cerebral e foi treinado pela desenvolvedora original do Sports Stars (GLC) em quatro reuniões online; o interventor local então treinou os outros membros da equipe antes do início do estudo. Detalhes das sessões do Sports Stars Brasil e exemplos de casos podem ser encontrados no protocolo publicado do estudo^22^ e em um estudo anterior.^12^ Um exemplo de sessão também está disponível na Figura 1.

#### Fisioterapia usual

Os participantes do grupo controle receberam o atendimento de fisioterapia usual. No Brasil, isso geralmente inclui intervenções como treino de habilidades motoras grossas, fortalecimento muscular e treino de equilíbrio.^19^ Pretendíamos coletar informações sobre a fisioterapia usual dos participantes (por exemplo, metas terapêuticas, características da intervenção, plano de tratamento, dosagem e adesão) entrando em contato direto com os fisioterapeutas das crianças. No entanto, os terapeutas não colaboraram fornecendo informações sobre o atendimento usual. Assim, as informações sobre o grupo de fisioterapia usual basearam‐se nas recordações dos pais e cuidadores, conforme descrito na Tabela S1. Segundo os pais e cuidadores, essas intervenções focavam principalmente na melhora do equilíbrio, coordenação e alinhamento das crianças durante a execução de habilidades fundamentais (por exemplo, caminhar, correr e controle de objetos) em ambiente clínico. As sessões incluíam exercícios voltados para força, equilíbrio, mobilidade e coordenação, além do treino em esteira. Durante o estudo, todas as crianças receberam uma ou duas sessões individuais de fisioterapia por semana (majoritariamente de 45 minutos cada), principalmente em instituições públicas. Este grupo recebeu um total de 12 a 24 horas de terapia, ao longo de 8 semanas. Após a realização da avaliação de follow‐up de 12 semanas, as crianças deste grupo controle foram convidadas a participar da intervenção de esportes modificados de 8 semanas, após o término do estudo, apenas por razões éticas.

### Características da amostra

A idade, sexo, tipo de PC (espástica, discinética, atáxica ou mista), distribuição (unilateral ou bilateral), nível do GMFCS^1^ e classificação da função manual (Manual Ability Classification System)^24^ foram coletados pelos pesquisadores no momento da entrada no estudo. Também coletamos a frequência (dias por semana) da participação em atividades físicas de lazer, incluindo educação física escolar e atividades físicas comunitárias. Além disso, a seção Ambiente Comunitário do *Participation and Environmental Measure for Children and Youth‐*PEM‐CY foi utilizada para caracterizar os ambientes dos participantes. Esse questionário fornece a porcentagem de apoio ambiental e barreiras, conforme aspectos como tempo, recursos ou dinheiro que podem dificultar a participação das crianças.^25^

### Medidas de desfecho

A Tabela 1 descreve as medidas de desfecho utilizadas. Detalhes adicionais sobre essas medidas e os procedimentos de aplicação podem ser encontrados no protocolo do estudo publicado.^22^ A coleta de dados ocorreu na linha de base, após a intervenção e no follow‐up de 12 semanas, conforme também realizado no estudo original do Sports Stars.^22^

**TABELA 1:** Desfechos primários e secundários.

		Instrumento/questionário	Descrição e procedimentos
**Desfecho primário**			
Comparecimento (isto é, a frequência com que a criança participa ou a variedade/diversidade de atividades^17^) e envolvimento (isto é, o engajamento, a motivação, a confiança ou a conexão social^17^) na participação em atividades físicas no tempo livre.		COPM	A COPM é uma medida de resultado centrada na pessoa. Nesse questionário semiestruturado, a criança e/ou sua família classificam e avaliam metas de desempenho e satisfação em uma escala de 10 pontos.^23^ Para este estudo, foi realizada uma administração modificada, conforme Clutterbuck et al.,^11^ na qual pais ou cuidadores escolheram duas metas no domínio de lazer da COPM: uma relacionada ao comparecimento (por exemplo, “Participar das aulas de educação física na escola duas vezes por semana” ou “Passar mais tempo durante o dia praticando brincadeiras de pular e correr”) e outra relacionada ao envolvimento em uma meta de atividade física de lazer (por exemplo, “Estar confiante e engajado ao participar de atividades de corrida com os amigos” ou “Ficar menos frustrado ao cometer erros ou perder em jogos com os amigos”).
**Desfechos secundários**			
Habilidades motoras	Desempenho	COPM	Uma terceira meta na administração modificada da COPM, definida pelos pais/cuidadoras, estava relacionada ao desempenho das crianças em habilidades motoras (por exemplo, correr, pular, chutar ou arremessar).
	Capacidade	TGMD‐2 e Challenge Test	O TGMD‐2 avalia os padrões de movimento durante a execução de habilidades motoras fundamentais.^26^ O Challenge Test mede 25 itens com habilidades motoras avançadas, como precisão e velocidade ao correr, pular ou arremessar, avaliando a capacidade de crianças deambuladoras com PC.^27^
Funções do corpo	Potência muscular e agilidade	MPST e 10X5ST	O 10X5ST é uma medida de agilidade e capacidade anaeróbica para indivíduos deambuladores com PC.^28^ O MPST avalia a potência muscular.
	Equilíbrio	Kids‐Mini‐BESTest	O Kids‐Mini‐BESTest é um teste padronizado de equilíbrio que possui 16 itens de ajustes posturais antecipatórios, respostas posturais reativas, orientação sensorial e estabilidade na marcha.^29^
Physical activity levels	Porcentagem de comportamento sedendário	Accelerometer (ActiGraph wGT3X‐BT, ActiGraph, Pensacola, FL, USA)	Trata‐se de um dispositivo pequeno e não invasivo que captura a magnitude da aceleração do tronco em três planos.^30^,^31^ Os participantes utilizam o dispositivo em um cinto elástico situado acima do quadril dominante. Nos três momentos deste estudo, os dispositivos ActiGraph foram usados pelos participantes durante sete dias consecutivos (cinco dias úteis e dois finais de semana). O dispositivo foi utilizado durante o horário habitual de vigília e retirado durante o sono e atividades na água. Períodos de não uso (máximo de dois minutos consecutivos com contagem zero) foram automaticamente detectados pelo dispositivo (algoritmo de Troiano^31^). Os dispositivos foram devolvidos após o sétimo dia de cada ciclo de uso para extração dos dados no software ActiLife 6 (ActiGraph, LLC, Pensacola, Florida).
	Porcentagem de atividade física leve		
	Porcentagem de atividade física moderada a vigorosa		
	Média de tempo por dia em atividade física moderada a vigorosa		
Perfil de alfabetização física nos domínios físico, social, psicológico e cognitivo		QPAF	O QPAF é uma ferramenta nova e validada que avalia os domínios físico, social, cognitivo e psicológico, assim como seus componentes, de acordo com a percepção dos pais sobre a alfabetização física de seus filhos. Possui 22 itens pontuados em uma escala de desempenho com 3 níveis.^32^
Participação geral		PEM‐CY	A PEM‐CY é um questionário preenchido pelos pais que mede a frequência, o envolvimento e o desejo de mudança na participação em três ambientes diferentes: casa, escola e comunidade.^25^

Abreviações: 10X5ST, teste de sprint de 10 corridas em 5 metros; COPM, Canadian Occupational Performance Measure; Kids‐Mini‐BESTest, Kids Mini Balance Evaluation Systems Test; MPST, Muscle Power Sprint Test; PEM‐CY, Participation and Environment Measure for Children and Youth; QPAF, Questionário Perfil de Alfabetização Física; TGMD‐2, Test of Gross Motor Development, Second Edition.

### Procedimentos

As medidas de desfecho foram aplicadas por dois fisioterapeutas cegados, formalmente treinados por pesquisadores seniores. Os testes físicos foram divididos em duas sessões dentro de uma semana: (1) Teste de Desenvolvimento Motor Grosso, Segunda Edição (TGMD‐2), *Muscle Power Sprint Test*, 10x5 *Sprint Test*, e Kids *Mini Balance Evaluation Systems Test* (Kids‐Mini‐BESTest) (administrados pelo avaliador 1) ou (2) *Challenge Test* (administrado pelo avaliador 2), para evitar o excesso de fadiga nas crianças. Pais/responsáveis responderam aos questionários online (exceto o COPM). Os avaliadores prestaram assistência em caso de dificuldades.

Pais/responsáveis foram convidados a observar todas as avaliações físicas dos seus filhos, e o COPM foi realizado presencialmente pelo avaliador 1. Nesse momento, o examinador pediu aos pais e responsáveis que escolhessem as metas de participação, dando exemplos e ajudando‐os a estruturar as metas. As crianças foram incentivadas a participar desse processo junto com suas famílias, expressando suas preferências.

Os participantes usaram dispositivos ActiGraph por 7 dias consecutivos (5 dias úteis e 2 dias de final de semana) antes das avaliações. O dispositivo foi utilizado durante as horas habituais de vigília e removido durante o sono e atividades aquáticas. Períodos de não uso (máximo de 2 minutos consecutivos com contagem zero) foram automaticamente detectados pelo dispositivo (algoritmo Troiano).^30^ Os dispositivos foram devolvidos após o 7° dia em cada ciclo de uso para extração dos dados no software ActiLife 6 (ActiGraph, Pensacola, FL, EUA). Todas as avaliações e definição de metas foram realizadas antes da randomização. A adesão à intervenção Sports Stars Brasil e todos os efeitos adversos foram documentados pelo pesquisador principal da intervenção.

### Adesão ao protocolo publicado do estudo

Este estudo foi conduzido conforme o protocolo publicado,^22^ com pequenas exceções conforme descrito a seguir. Em relação ao recrutamento, nosso protocolo original também incluía adolescentes; entretanto, o estudo foi limitado a crianças de 6 a 12 anos devido à dificuldade em recrutar adolescentes com PC na nossa região. Um estudo piloto com adolescentes com PC está em andamento. Quanto às medidas de desfecho, o Questionário Perfil de Alfabetização Física foi identificado pelo nome inicial em nosso artigo de protocolo (‘Percepções de pais/responsáveis nos domínios da alfabetização física’), pois estava em desenvolvimento na época da publicação do protocolo. Não foi possível coletar a frequência de participação das crianças (média de minutos por dia) em esportes e atividades recreativas físicas, conforme planejado, porque os pais não cumpriram a tarefa de preencher os registros diários. Para avaliar os níveis de atividade física por acelerometria, optamos por substituir os pontos de corte gerais mencionados em nosso protocolo^22^ pelos descritos por Trost et al.^30^, pois são específicos para crianças nos níveis I e II do GMFCS. Por fim, planejamos avaliar as proporções de participantes que alcançaram a diferença mínima clinicamente importante para os escores do COPM, considerando uma mudança maior que 2 pontos como clinicamente significativa. No entanto, como evidências atuais refutam os valores mínimos de diferença clinicamente importante para o COPM^33^, decidimos não prosseguir com essa análise.

### Análise estatística

Estatísticas descritivas resumiram as características dos participantes (idade, sexo, classificação da PC, características sociodemográficas). Os dados foram apresentados como média e desvio padrão. A normalidade dos dados dos desfechos foi investigada pelo teste de Shapiro–Wilk. As diferenças basais entre os grupos foram avaliadas pelo teste t independente ou teste *Mann–Whitney U*. Modelos lineares mistos^34^ foram utilizados para analisar os desfechos primários e secundários, comparando o grupo Sports Stars e o grupo de fisioterapia usual após a intervenção e no follow‐up. Os modelos incluíram interceptos aleatórios e coeficientes fixos, incorporando os grupos de tratamento, os momentos de avaliação (baseline, pós‐intervenção e follow‐up) e as interações tratamento × tempo. Os resultados foram apresentados como diferenças de médias com intervalos de confiança de 95% (IC) e tamanhos de efeito de Cohen (d). Os tamanhos de efeito foram interpretados segundo os critérios de Cohen: efeitos pequenos (<0,14), moderados (0,30–0,60) e grandes (>0,60).^35^ Todas as análises estatísticas foram conduzidas no software Statistical Package for the Social Sciences (SPSS), versão 26 (IBM Corp., Armonk, NY, EUA).

## RESULTADOS

Trinta e oito crianças foram recrutadas e avaliadas na linha de base. As características demográficas dos participantes estão apresentadas na Tabela 2. Os grupos foram semelhantes em suas características demográficas e ambientais, frequência de participação em atividades físicas e em todas as medidas de desfecho primário e secundário na linha de base. Não houve desistências após a randomização ou durante o estudo em nenhum dos grupos. No entanto, houve de três a sete casos com dados ausentes em algumas avaliações pós‐intervenção e de seguimento (ver o diagrama de fluxo CONSORT na Figura S1 com os números e as principais razões).

**TABELA 2:** Características dos participantes.

	Fisioterapia usual (*n* = 19)	Sports Stars Brasil (*n* = 19)
**Características dos participantes**		
Média de idade	8 anos e 4 meses (DP 1 ano e 11 meses, mínimo 8 anos, máximo 11 anos)	8 anos e 11 meses (DP 1 ano e 9 meses, mínimo 6 anos, máximo 12 anos)
Tipo de PC	Espástica: 18 (94%) Atáxica: 1 (6%) Mista:0 (0%)	Espástica:18 (94%) Atáxica:0 (0%) Mista:1 (6%)
Sexo biológico	Masculino: 10 (53%) Feminino: 9 (47%)	Masculino: 11 (57%) Feminino: 8 (43%)
Topografia da PC	Unilateral: 12 (63%) Bilateral: 7 (34%)	Unilateral: 9 (47%) Bilateral: 10 (53%)
GMFCS	I: 13 (68%) II: 6 (32%)	I: 13 (68%) II: 6 (32%)
MACS	I:13 (68%) II: 4 (21%) III: 2 (11%)	I: 12 (63%) II:6 (32%) III: 1 (5%)
Frequência de atividade física no baseline	0 dias: 17 (89.48%) 1 dia: 2(10.52%) 2 dias: 0	0 dias: 16 (84.21%) 1 dias: 1 (5.3%) 2 dias: 2(10.52%)
**Ambiente da comunidade no baseline‐ Medida pela PEM‐CY**		
Apoios	60.57% (DP 6.19)	60.94% (DP 5.21)
Barreiras	39.25% (DP 6.20)	38.84% (DP 5.22)
Facilitadores	86.10% (DP 3.55)	89.00% (DP 2.32)
Recursos	78.42% (DP 2.85)	78.84% (DP 3.37)
Apoio geral	82.84% (DP 2.72)	82.10% (DP 2.50)

Abreviações: PC, paralisia cerebral; GMFCS, Gross Motor Function Classification System; MACS, Manual Ability Classification System; PEM‐CY, Participation and Environment Measure for Children and Youth; DP, desvio padrão.

### Adesão e efeitos adversos

A média de presença no grupo Sports Stars Brasil foi de 6,68 sessões (DP 1,29) das oito sessões (83,5%) ao longo das 8 semanas. Apenas um participante desse grupo relatou um evento adverso leve, apresentando dor muscular na panturrilha após o exercício no primeiro dia de intervenção, que se resolveu um dia depois e não voltou a ocorrer. O grupo de fisioterapia usual participou de uma ou duas sessões de fisioterapia por semana durante as 8 semanas (média de 2 dias por semana, DP 0,75). Embora não tenhamos conseguido coletar dados reais sobre adesão (percentual de sessões frequentadas) ao tratamento usual de fisioterapia na perspectiva dos fisioterapeutas das crianças, os pais relataram que as crianças tiveram poucas ausências durante o período do estudo.

### Resultados primários

A Tabela 3 apresenta a lista de todas as metas do COPM, escolhidas por pais e cuidadores na linha de base. Essas metas, definidas pelos pais, nos grupos de intervenção e fisioterapia usual apresentaram conteúdo semelhante.

**TABELA 3:** Metas de participação (frequência/envolvimento em atividades físicas) e desempenho motor definidas por pais e cuidadores.

Comparecimento em atividades físicas de lazer	Envolvimento em atividades físicas de lazer	Desempenho em atividades motoras fundamentais
**Grupo Sports Stars Brasil** “Brincar de pique mais vezes com os amigos em casa” “Jogar futebol com os primos com mais frequência” “Passar mais tempo praticando corrida” “Começar a praticar natação pelo menos 2 vezes por semana” “Começar a praticar esportes favoritos (basquete, ginástica) 1 a 2 vezes por semana” “Passar mais tempo durante o dia praticando jogos de pular e correr” “Começar a praticar um esporte (por exemplo, natação) pelo menos uma vez por semana” (duas crianças) “Jogar mais vezes jogos com bola” (duas crianças) “Participar mais das aulas de educação física na escola” (três crianças) “Jogar futebol por mais tempo com os amigos” (três crianças) “Começar a praticar um esporte pelo menos uma vez por semana” (três crianças) **Grupo Fisioterapia usual** “Passar mais tempo durante o dia praticando jogos de pular e correr” “Brincar com bola com a irmã com mais frequência durante a semana” “Brincar no trampolim com mais frequência durante a semana” “Começar a jogar futebol com os amigos” (duas crianças) “Jogar futebol por mais tempo com os amigos” (duas crianças) “Participar mais das aulas de educação física na escola” (quatro crianças) “Começar a praticar natação pelo menos uma vez por semana” (três crianças) “Começar a praticar um esporte pelo menos duas vezes por semana” (três crianças) “Começar a praticar um esporte pelo menos uma vez por semana” (duas crianças)	**Grupo Sports Stars Brasil** “Estar mais motivado para jogar futebol com os primos” “Estar mais confiante em atividades que envolvem equilíbrio, como correr e pular” “Estar mais envolvido nos jogos no parque nos finais de semana” “Ter mais segurança e confiança para praticar natação” “Ter mais confiança para mudar de direção ao andar de bicicleta com os pais” “Sentir‐se mais seguro e confiante para brincar com os colegas” “Estar mais focado durante os jogos de futebol” (duas crianças) “Saber lidar com derrotas (ficar confiante e menos frustrado) enquanto joga com os amigos” (duas crianças) “Sentir‐se mais confiante para participar de jogos com outras crianças” (três crianças) “Ter mais foco durante os jogos com os amigos” (três crianças) “Estar mais engajado durante a participação em atividades físicas” (três crianças) **Grupo Fisioterapia usual** “Ter mais confiança para jogar futebol com outras crianças” “Sentir menos medo de participar de atividades com bola” “Ficar menos frustrado ao cometer erros ou perder durante jogos com amigos” “Ter mais segurança e confiança para brincar com crianças desconhecidas” “Ter menos medo de cair enquanto corre e brinca com outras crianças” “Sentir‐se mais confiante para participar de jogos com outras crianças” (duas crianças) “Sentir‐se mais seguro e confiante para brincar com colegas” (duas crianças) “Sentir‐se mais confiante para participar de atividades físicas na escola” (duas crianças) “Ter mais foco durante jogos com amigos” (três crianças) “Estar mais engajado durante a participação em atividades físicas” (cinco crianças)	**Grupo Sports Stars Brasil** “Pular mais alto” “Correr com mais coordenação” “Pegar bolinhas pequenas” “Conseguir quicar uma bola de basquete com as duas mãos” “Arremessar bolas com as duas mãos” “Pegar a bola em movimento” / “Quicar uma bola de basquete” (duas crianças) “Chutar uma bola” (duas crianças) “Conseguir arremessar uma bola” (três crianças) “Correr e chutar a bola sem parar” (três crianças) “Correr com mais equilíbrio” (três crianças) **Grupo fisioterapia usual** “Correr com melhor qualidade” “Chutar com a perna afetada” “Saltar com o pé afetado” “Correr e chutar a bola sem parar” “Chutar a bola de futebol com direção” “Arremessar a bola com as duas mãos” “Conseguir pular em um pé só com mais equilíbrio” “Correr e chutar a bola sem parar” (duas crianças) “Conseguir arremessar a bola” (três crianças) “Correr com mais coordenação” (três crianças) “Chutar a bola” (quatro crianças)

A Tabela 4 exibe os resultados dos desfechos primários comparando ambos os grupos. Imediatamente após 8 semanas de intervenção, o Sports Stars Brasil foi mais eficaz do que a fisioterapia usual na melhora das metas relacionadas à frequência em atividades físicas de lazer (COPM desempenho: diferença média = 2,05 [0,42–3,68], d = 0,97; COPM satisfação: diferença média = 2,05 [0,23–3,87], d = 0,80) e envolvimento (COPM desempenho: diferença média = 1,84 [0,67–3,01], d = 0,65; COPM satisfação: diferença média = 2,10 [0,51–3,69], d = 1,01). A magnitude das diferenças entre Sports Stars Brasil e fisioterapia usual nas melhorias das metas relacionadas à atividade física de lazer foi geralmente alta. A superioridade do Sports Stars Brasil foi mantida no seguimento de 12 semanas, apenas para a meta de frequência (COPM desempenho: diferença média = 1,85 [0,11–3,58], d = 0,80; COPM satisfação: diferença média = 1,72 [−0,21 a 3,67], d = 0,65), embora com tamanhos de efeito menores. As mudanças intra‐grupos ao longo do tempo estão apresentadas na Tabela S2.

**TABELA 4:** Comparações entre grupos Sports Stars Brasil (n = 19) e fisioterapia usual (*n* = 19) para escores totais do COPM.

Metas de participação medidas pelo COPM (unidade: pontos de escore de 1 a 10)	Tempo	Sports Stars Brasil, média (DP)	Fisioterapia usua, média (DP)	Diferença media entre grupos (95% IC)	Tamanho de efeito
Comparecimento: desempenho	Baseline	3.00 (2.47)	2.33 (1.97)	—	—
	Pós‐intervenção	5.77 (3.42)	3.05 (2.53)	2.05 (0.42 to 3.68)	0.97
	Follow‐up de 12 semanas	6.33 (3.43)	3.73 (2.98)	1.85 (0.11 to 3.58)	0.80
Comparecimento: satisfação	Baseline	3.16 (3.05)	2.55 (2.40)	—	—
	Pós‐intervenção	6.16 (3.71)	3.50 (3.24)	2.05 (0.23 to 3.87)	0.85
	Follow‐up de 12 semanas	6.60 (3.52)	4.26 (3.59)	1.72 (−0.21 to 3.67)	0.65
Envolvimento: desempenho	Baseline	4.94 (1.80)	5.31 (1.85)	—	—
	Pós‐intervenção	7.15 (2.33)	5.68 (2.18)	1.84 (0.67 to 3.01)	0.65
	Follow‐up de 12 semanas	7.25 (2.48)	6.75 (1.73)	0.86 (−0.38 to 2.10)	0.23
Envolvimento: satisfação	Baseline	4.94 (2.50)	4.52 (2.29)	—	—
	Pós‐intervenção	7.73 (2.28)	5.21 (2.67)	2.10 (0.51 to 3.69)	1.01
	Follow‐up de 12 semanas	8.00 (2.25)	6.43 (2.36)	1.04 (−0.63 to 2.72)	0.68

Abreviações: IC, interval de confiança; COPM, Canadian Occupational Performance Measure; DP, desvio padrão.

### Resultados secundários

A Tabela 5 apresenta os resultados dos desfechos secundários. O Sports Stars Brasil foi mais eficaz do que a fisioterapia usual na melhora das metas relacionadas ao desempenho motor (COPM desempenho: diferença média = 2,21 [1,23–3,18], d = 0,80; COPM satisfação: diferença média = 2,47 [1,27–3,66], d = 0,96). A magnitude da diferença entre Sports Stars Brasil e fisioterapia usual nas melhorias das metas de desempenho motor foi alta. Esses efeitos foram sustentados no seguimento de 12 semanas (COPM desempenho: diferença média = 2,17 [1,14–3,21], d = 0,67; COPM satisfação: diferença média = 2,14 [0,87–3,41], d = 0,89), com tamanhos de efeito moderados a altos.

**TABELA 5:** Comparação dos escores entre os grupos Sports Stars Brasil (n = 19) e fisioterapia usual (*n* = 19): análise entre grupos das medidas de desfechos secundários.

	Tempo	Sports Stars Brasil, média (DP)	Fisioterapia usua, média (DP)	Diferença media entre grupos (95% IC)	Tamanho de efeito
Desempenho motor: desempenho medidas pelo COPM Unidade: pontos de escore de 1 a 10	Baseline	4.57 (1.74)	5.26 (1.66)	—	—
	Pós‐intervenção	7.21 (1.96)	5.68 (1.84)	2.21 (1.23 to 3.18)	0.80
	Follow‐up de 12 semanas	7.71 (1.82)	6.06 (1.87)	2.17 (1.14 to 3.21)	0.67
Desempenho motor: satisfação medidas pelo COPM Unidade: pontos de escore de 1 a 10	Baseline	5.47 (2.16)	5.68 (2.23)	—	—
	Pós‐intervenção	8.31 (2.10)	6.05 (2.57)	2.47 (1.27 to 3.66)	0.96
	Follow‐up de 12 semanas	8.56 (1.89)	6.50 (2.65)	2.14 (0.87 to 3.41)	0.89
Capacidade das habilidades motoras de locomoção Medida pelo TGMD‐2 Unidade: pontos 0–48	Baseline	29.15 (10.43)	31.22 (9.34)	—	—
	Pós‐intervenção	32.33 (9.57)	37.38 (9.81)	—2.06 (−7.04 to 8.78)	0.20
	Follow‐up de 12 semanas	34.66 (9.16)	32.16 (9.90)	2.44 (−2.56 to 5.23)	0.22
Capacidade das habilidades motoras de controle de objetos Medida pelo TGMD‐2 Unidade: pontos 0–48	Baseline	28.40 (10.67)	31.23 (8.73)	—	—
	Pós‐intervenção	34.55 (7.42)	33.23 (6.98)	4.29 (−0.47 to 9.07)	0.18
	Follow‐up de 12 semanas	38.00 (5.73)	33.46 (8.15)	6.80 (1.82 to 11.78)	0.64
Capacidade das habilidades motoras no geral Medida pelo TGMD‐2 Unidade: pontos 0–96	Baseline	57.55 (18.72)	65.05 (17.15)	—	—
	Pós‐intervenção	66.88 (15.86)	70.61 (16.00)	2.08 (−5.83 to 10.00)	0.23
	Follow‐up de 12 semanas	72.92 (13.68)	65.08 (16.53)	9.00 (0.61 to 17.40)	0.51
Capacidade das habilidades motoras Medida pelo Challenge Test Unidade: pontos em porcentagem	Baseline	35.84 (20.10)	40.55 (21.23)	—	—
	Pós‐intervenção	41.40 (21.57)	47.57 (24.67)	—3.32 (−8.04 to 1.39)	0.26
	Follow‐up de 12 semanas	42.72 (19.10)	42.43 (24.23)	0.52 (−5.42 to 4.36)	0.01
Níveis de atividade física: comportamento sedentário Medido pelo acelerômetro Unidade: tempo em porcentagem	Baseline	59.45 (13.73)	54.48 (9.94)	—	—
	Pós‐intervenção	57.29 (11.86)	57.01 (8.60)	—2.05 (−6.97 to 2.87)	0.02
	Follow‐up de 12 semanas	55.52 (10.97)	55.73 (10.45)	—3.79 (−8.66 to 1.06)	0.01
Níveis de atividade física: atividade física leve Medido pelo acelerômetro Unidade: tempo em porcentagem	Baseline	30.11 (10.01)	33.62 (5.70)	—	—
	Pós‐intervenção	31.52 (8.09)	32.07 (5.06)	1.25 (−2.00 to 4.52)	0.08
	Follow‐up de 12 semanas	32.19 (7.72)	33.34 (8.06)	1.29 (−1.92 to 4.52)	0.14
Níveis de atividade física: atividade física moderada a vigorosa Medido pelo acelerômetro Unidade: tempo em porcentagem	Baseline	10.37 (4.35)	11.87 (3.46)	—	—
	Pós‐intervenção	11.17 (4.58)	10.86 (4.11)	1.00 (−1.59 to 3.60)	0.07
	Follow‐up de 12 semanas	12.18 (4.78)	10.53 (4.88)	2.89 (0.33 to 5.46)	0.34
Níveis de atividade física: atividade física moderada a vigorosa por semana Medido pelo acelerômetro Unidade: média de minutos	Baseline	65.63 (28.68)	69.05 (20.64)	—	—
	Pós‐intervenção	64.29 (32.81)	61.53 (21.89)	2.51 (13.69 to 18.72)	0.09
	Follow‐up de 12 semanas	68.56 (31.32)	51.76 (20.64)	15.59 (−0.42 to 31.60)	0.63
Alfabetização física Medida pelo QPAF Unidade: pontos em porcentagem	Baseline	69.94 (15.79)	70.45 (17.29)	—	—
	Pós‐intervenção	81.69 (15.02)	75.43 (15.98)	6.76 (−3.35 to 16.88)	0.40
	Follow‐up de 12 semanas	79.05 (19.12)	72.05 (20.99)	6.56 (−4.43 to 17.00)	0.34
Equilíbrio Medido pelo Kids‐Mini‐BESTest Unidade: pontos 0–34	Baseline	26.25 (4.20)	27.52 (2.83)	—	—
	Pós‐intervenção	25.56 (6.98)	28.38 (2.46)	—1.05 (−3.39 to 1.29)	0.53
	Follow‐up de 12 semanas	26.73 (3.57)	27.23 (3.39)	0.02 (−2.34 to 2.39)	0.14
Potência muscular: média Medida pelo MPST Unidade: potência (watts)	Baseline	69.02 (39.56)	77.40 (49.78)	—	—
	Pós‐intervenção	75.23 (41.96)	79.19 (66.57)	3.05 (−10.91 to 17.02)	0.07
	Follow‐up de 12 semanas	73.41 (40.56)	56.19 (42.43)	12.67 (−1.46 to 26.80)	0.41
Potência muscular: pico Medida pelo MPST Unidade: potência (watts)	Baseline	81.55 (47.61)	95.32 (64.17)	—	—
	Pós‐intervenção	93.33 (51.68)	94.09 (72.84)	10.75 (−6.05 to 27.56)	0.01
	Follow‐up de 12 semanas	91.36 (44.62)	79.72 (59.98)	10.65 (−6.36 to 27.66)	0.22
Agilidade Medida pelo 10X5ST Unidade: segundos	Baseline	36.49 (12.51)	36.03 (11.95)	—	—
	Pós‐intervenção	39.16 (19.79)	35.26 (8.68)	0.85 (−3.19 to 4.89)	0.25
	Follow‐up de 12 semanas	35.37 (6.26)	37.20 (7.95)	1.16 (−5.33 to 3.00)	0.25
Participação geral: frequência na escola Medida pela PEM‐CY Unidade: pontos 0–7	Baseline	5.36 (1.53)	5.15 (1.80)	—	—
	Pós‐intervenção	5.26 (1.28)	4.84 (1.64)	0.21 (−0.70 to 1.12)	0.28
	Follow‐up de 12 semanas	5.33 (1.23)	5.26 (1.38)	0.10 (−1.10 to 0.88)	0.05
Participação geral: envolvimento na escola Medida pela PEM‐CY Unidade: pontos 0–5	Baseline	4.42 (0.76)	3.89 (1.24)	—	—
	Pós‐intervenção	4.47 (0.61)	4.15 (1.21)	−0.21 (−0.73 to 0.30)	0.33
	Follow‐up de 12 semanas	4.60 (0.73)	4.33 (0.89)	−0.19 (−0.75 to 0.37)	0.33
Participação geral: desejo de mudança na escola Medida pela PEM‐CY Unidade: porcentagem	Baseline	64.21 (34.19)	71.57 (37.03)	—	—
	Pós‐intervenção	55.78 (35.63)	63.15 (34.80)	0.00 (−20.74 to 20.74)	0.20
	Follow‐up de 12 semanas	54.66 (34.19)	54.66 (26.69)	6.85 (−15.66 to 29.39)	0.01
Participação geral: número de atividades na escola Medida pela PEM‐CY Unidade: porcentagem	Baseline	3.57 (1.12)	3.00 (1.41)	—	—
	Pós‐intervenção	3.94 (1.12)	3.52 (1.42)	−0.15 (−1.00 to 0.68)	0.44
	Follow‐up de 12 semanas	3.60 (1.24)	3.60 (1.40)	−0.53 (−1.45 to 0.38)	0.01
Participação geral: frequência na comunidade Medida pela PEM‐CY Unidade: pontos 0–7	Baseline	4.42 (1.16)	4.42 (1.26)	—	—
	Pós‐intervenção	4.26 (1.34)	4.26 (1.09)	0.15 (−0.47 to 0.89)	0.01
	Follow‐up de 12 semanas	4.40 (1.12)	4.40 (1.05)	0.21 (−0.59 to 0.90)	0.01
Participação geral: envolvimento na comunidade Medida pela PEM‐CY Unidade: pontos 0–5	Baseline	4.31 (0.67)	4.47 (0.69)	—	—
	Pós‐intervenção	4.52 (0.77)	4.63 (0.68)	−0.15 (−0.45 to 0.35)	0.15
	Follow‐up de 12 semanas	4.60 (0.63)	4.60 (0.68)	−0.05 (−0.60 to 0.28)	0.01
Participação geral: desejo de mudança na comunidade Medida pela PEM‐CY Unidade: porcentagem	Baseline	49.33 (28.65)	62.63 (28.75)	—	—
	Pós‐intervenção	64.21 (25.45)	56.31 (30.40)	3.70 (−9.79 to 17.20)	0.28
	Follow‐up de 12 semanas	68.84 (24.87)	44.66 (29.97)	2.01 (−11.48 to 15.52)	0.29
Participação geral: número de atividades na comunidade Medida pela PEM‐CY Unidade: porcentagem	Baseline	5.52 (2.31)	5.57 (2.09)	—	—
	Pós‐intervenção	6.15 (2.00)	5.94 (2.06)	0.26 (−0.74 to 1.46)	0.09
	Follow‐up de 12 semanas	5.86 (2.16)	6.46 (2.16)	−0.33 (−1.43 to 0.75)	0.27

Abreviações: 10X5ST, 10 corridas por 5 metros; IC, interval de confiança; COPM, Canadian Occupational Performance Measure; Kids‐Mini‐BESTest, Kids Mini Balance Evaluation Systems Test; MPST, Muscle Power Sprint Test; PEM‐CY, Participation and Environment Measure for Children and Youth; QPAF, Questionário Perfil de Alfabetização Física, DP, desvio padrão; TGMD‐2, Test of Gross Motor Development, Second Edition.

Além disso, o Sports Stars Brasil superou a fisioterapia usual na capacidade de habilidades motoras (TGMD‐2 controle de objetos: diferença média = 6,80 [1,82–11,78], d = 0,64; TGMD‐2 total: diferença média = 9,00 [0,61–17,40], d = 0,51) e na porcentagem de tempo gasto em atividade física de intensidade moderada a vigorosa no seguimento de 12 semanas (diferença média = 2,89 [0,33–5,46], d = 0,34), com tamanhos de efeito moderados. Diferenças com tamanhos de efeito pequenos foram observadas nos demais desfechos. As mudanças intragrupos ao longo do tempo estão apresentadas na Tabela S2.

## DISCUSSÃO

O principal objetivo deste ensaio clínico randomizado foi avaliar a efetividade do Sports Stars Brasil para crianças com PC nos níveis I e II do GMFCS. Comparado ao grupo de fisioterapia usual, que recebeu sessões uma ou duas vezes por semana, essa intervenção de esportes modificados em grupo, conduzida por profissionais, mostrou‐se superior na melhora dos objetivos de participação em atividades físicas de lazer relacionados à frequência e ao envolvimento, assim como no desempenho de habilidades motoras. Além disso, o Sports Stars Brasil foi similar à fisioterapia usual na melhora da capacidade motora e da alfabetização física neste grupo de crianças. Nosso estudo contribui para o crescente corpo de evidências que apoia os esportes modificados como intervenção de reabilitação para crianças com PC, especialmente em países de baixa e média renda, como o Brasil. A seguir, discutimos os resultados deste estudo à luz dos componentes do tratamento com esportes modificados.

De forma semelhante ao programa original Sports Stars na Austrália, os maiores escores do COPM para ganhos em participação em atividades físicas de lazer ocorreram após a intervenção e foram mantidos no follow‐u^13^. Acreditamos que, neste estudo, as diferenças nos objetivos de participação — frequência e envolvimento — favorecendo o Sports Stars Brasil após o término da intervenção, podem ser atribuídas ao fato de que essa intervenção incluiu ingredientes ativos relacionados a todos os domínios da alfabetização física (físico, cognitivo, social e psicológico), desenvolvendo habilidades para alcançar os objetivos de participação das crianças. Em contraste, crianças no grupo de fisioterapia usual podem ter se envolvido principalmente com o domínio físico (Tabela S1). Segundo revisões anteriores que avaliaram a efetividade de intervenções focadas na participação, fatores contextuais — incluindo características pessoais das crianças — devem ser considerados ao se buscar melhorar a participação em diferentes contextos^36^. No contexto das atividades físicas de lazer, esses fatores pessoais estão intimamente ligados aos domínios da alfabetização física^8^. Nossos achados reforçam a ideia de que melhorar a frequência e o envolvimento em atividades físicas exige uma compreensão abrangente da alfabetização física da criança. Acreditamos que o Sports Stars Brasil promoveu habilidades de alfabetização física para melhorias adicionais na participação em atividades físicas de lazer (ou seja, participação em esportes ou recreação física).

Considerando os resultados relacionados aos objetivos de frequência e envolvimento, acreditamos que o Sports Stars Brasil está alinhado à Família de Constructos de Participação de Imms et al.^17^, na qual frequência e envolvimento estão relacionados a fatores intrínsecos (senso de si, preferências e competência na atividade) e extrínsecos (ambiente). Como observado em nosso estudo qualitativo anterior^12^, o programa Sports Stars não apenas melhora a competência na atividade das crianças por meio do desenvolvimento de habilidades motoras, mas também fortalece sua autoconfiança (senso de si) e desperta interesse por atividades físicas (preferências). Essas oportunidades de prática em ambientes reais com colegas, aliadas às demandas sociais, cognitivas e psicológicas, englobam a maioria dos constructos de participação, fornecendo um contexto favorável para promover atividades físicas de lazer. Acreditamos ser essencial considerar esses ingredientes ao promover a participação em atividades físicas de lazer. Incluir tais elementos garante que uma intervenção de esportes modificados esteja alinhada à sua definição: “Programas de esportes modificados são oferecidos para engajar crianças em atividades lúdicas projetadas, entre outras coisas, para desenvolver habilidades motoras fundamentais e específicas para esportes, com vistas à participação futura”.^6^

Embora o grupo de fisioterapia usual tenha recebido consideravelmente mais intervenção (12–24 horas de terapia versus 8 horas), o Sports Stars Brasil foi mais efetivo na promoção do desempenho motor das crianças, conforme os objetivos de desempenho do COPM (isto é, em ambientes da vida cotidiana). Ambos os grupos apresentaram resultados semelhantes no pós‐intervenção quanto à capacidade motora, avaliada em ambientes controlados pelos testes Challenge e TGMD‐2. Embora as intervenções oferecidas ao grupo de fisioterapia usual não tenham sido formalmente padronizadas, pais e cuidadores relataram que as crianças desse grupo praticaram individualmente tarefas motoras grosseiras, treinamento de força e equilíbrio em ambiente clínico. Supomos que os fisioterapeutas tenham selecionado ingredientes ativos nessas intervenções para melhorar diretamente ou indiretamente as habilidades motoras das crianças.^37^ Isso pode explicar a principal diferença entre os grupos no desempenho motor e a semelhança na capacidade motora. Enquanto ambas as intervenções visaram a capacidade motora por meio de um ingrediente ativo comum — prática estruturada e repetida de tarefas —, os ingredientes adicionais necessários para melhorar o desempenho motor, como a prática em ambientes reais, provavelmente estavam presentes apenas no grupo Sports Stars Brasil. Como enfatizado por Jackman et al.^38^, a prática em contextos reais é essencial para que crianças com PC transfiram os ganhos para a vida diária. O Sports Stars pode ser uma opção de intervenção custo‐efetiva para promover desempenho e capacidade motora, ao produzir resultados similares aos da fisioterapia usual com menor tempo de intervenção.

O Sports Stars Brasil não foi efetivo na melhora dos níveis de atividade física, das funções corporais e da participação geral (avaliada pela PEM‐CY), nem imediatamente após nem 12 semanas após a intervenção. Hipotetizamos que mudanças nas funções corporais após intervenções com esportes modificados podem ser obtidas com programas de maior dosagem e duração (para produzir mudanças musculoesqueléticas efetivas), como observado em outros estudos com esportes modificados para crianças com PC.^39^, ^40^. Embora as crianças tenham participado mais de atividades físicas após o Sports Stars (conforme melhorias nos objetivos de participação do COPM), a magnitude dessa mudança foi pequena e insuficiente para promover alterações mais amplas no estilo de vida que reduzissem o comportamento sedentário logo após a intervenção. Como indicado por Reedman et al.^41^, intervenções que incluem aspectos motivacionais e de mudança de comportamento provavelmente abordam melhor os níveis de atividade física em indivíduos com PC. Programas futuros de esportes modificados combinados a estratégias para promover comportamentos saudáveis e motivação podem alcançar melhores resultados. Além disso, a ausência de mudanças na participação geral reforça achados anteriores de que intervenções voltadas à participação dificilmente geram ganhos generalizados nos contextos de casa, escola e comunidade.^8^, ^36^.

É importante destacar que apenas após 12 semanas de seguimento as crianças do grupo Sports Stars Brasil demonstraram maiores melhorias no tempo médio gasto em atividade física moderada a vigorosa por semana e na capacidade motora avaliada pelo TGMD‐2, especialmente no controle de objetos. Acreditamos que esses resultados sejam ganhos indiretos da intervenção. Ao participarem do programa, as crianças aumentaram sua frequência e envolvimento nas sessões de esporte e recreação. Como consequência, após 12 semanas, estavam mais ativas nos dias de semana e tiveram mais oportunidades para praticar suas habilidades motoras em programas de atividade física comunitária convencional, aprimorando seus padrões de movimento ao executar essas habilidades.

Diante do exposto, hipotetizamos que o Sports Stars Brasil possa ser uma opção economicamente mais viável para os gestores, especialmente em países de baixa e média renda como o Brasil. Comparada à fisioterapia tradicional individualizada para crianças deambuladoras com PC, essa intervenção em grupo, de baixa dosagem e baixo custo, demonstrou resultados superiores em participação e desempenho motor, e similares em capacidade motora. A intervenção Sports Stars exigiu menos tempo total (uma vez por semana, totalizando 8 horas em 2 meses) do que a fisioterapia usual (tipicamente duas vezes por semana, totalizando 12–24 horas no mesmo período). Além disso, pode ser aplicada com uma menor razão terapeuta–criança (um a quatro terapeutas para quatro a oito crianças simultaneamente), ao contrário da terapia tradicional, que costuma ser oferecida no formato individualizado. Esse formato pode facilitar a oferta de serviços de fisioterapia e habilitação para crianças deambuladoras com PC, especialmente em sistemas públicos de saúde. Estudos futuros de análise econômica poderão confirmar essa hipótese.

Estudos futuros poderiam investigar os parâmetros de dosagem apropriados de intervenções com esportes modificados para alcançar resultados secundários em funções corporais relevantes para crianças com PC. Além disso, considerando que a participação é fortemente influenciada pelo contexto, acreditamos que futuras pesquisas que combinem esportes modificados com abordagens centradas no contexto podem aumentar os níveis de atividade física das crianças, reduzir o comportamento sedentário e remover barreiras ambientais à participação em atividades físicas de lazer. Um estudo está atualmente em andamento para avaliar a viabilidade de combinar o Sports Stars Brasil com uma terapia centrada no contexto.^42^

Este estudo apresenta algumas limitações que merecem ser informadas. Uma delas é que, embora nosso tamanho amostral tenha sido adequado para o desfecho primário, o poder estatístico foi insuficiente (22–64%) para investigar diferenças em alguns dos desfechos secundários, como alfabetização física e os testes de capacidade motora. Estudos futuros com amostras maiores são necessários para investigar mais a fundo a efetividade de intervenções com esportes modificados nos desfechos secundários deste estudo. Além disso, este estudo comparou o Sports Stars com a fisioterapia usual; estudos futuros também devem comparar essa intervenção com terapias controladas específicas (por exemplo, treino de tarefas específicas). Também não conseguimos coletar dados reais sobre o grupo de fisioterapia usual, pois não foi possível entrar em contato com os fisioterapeutas das crianças, sendo necessário recorrer ao relato de pais e cuidadores. Informações sobre o tratamento e a adesão do grupo controle devem ser melhor coletadas em estudos futuros. Outra limitação potencial é o viés de seleção devido à perda de participantes durante o seguimento, pois houve perdas em alguns desfechos secundários. Por fim, devido à ausência de estudos de interpretabilidade da maioria dos instrumentos e questionários utilizados, não foi possível realizar a análise da proporção de participantes que atingiram diferenças clinicamente importantes.

## CONCLUSÃO

O Sports Stars Brasil foi eficaz na melhoria de metas relacionadas à participação em atividades físicas durante o tempo de lazer, especificamente quanto à frequência e ao envolvimento, entre crianças deambuladoras com paralisia cerebral. Essa intervenção aprimorou o desempenho motor dos participantes em habilidades motoras fundamentais, apresentando resultados comparáveis aos de programas usuais de atividade física no que se refere à capacidade motora e alfabetização física. Não foram observadas melhorias nos níveis de atividade física, funções do corpo ou na participação geral após o programa Sports Stars Brasil. Essa intervenção de esportes modificados é uma abordagem eficaz e de baixo custo, com potencial de implementação na prática clínica, especialmente em países de baixa e média renda.

### AGRADECIMENTOS

Agradecemos à Universidade Federal de Minas Gerais e ao Conselho Nacional de Desenvolvimento Científico e Tecnológico pelo apoio institucional. Este estudo contou com o apoio da Coordenação de Aperfeiçoamento de Pessoal de Nível Superior, na forma de bolsas de doutorado e de pesquisa.

## Supporting information


**Figure S1:** CONSORT diagram.

**Table S1:** Usual therapy group: Physical therapy information according to parents and caregivers.

**Table S2:** Changes over the time for Sports Stars Brasil and usual physical therapy groups.
