# Supplementary material for: Intervenção de esportes modificados para melhorar metas de participação e competências de atividade em crianças deambuladoras com paralisia cerebral: um ensaio clínico randomizado
Source: Dev Med Child Neurol. 2025 Jul 3;68(1):e1–e15. doi: 10.1111/dmcn.16411 (PMC12683299; doi:10.1111/dmcn.16411)
Supplement: Supplementary file 1 — Figure S1: CONSORT diagram. [file DMCN-68-e1-s003.pdf]

Machine Translated by Google

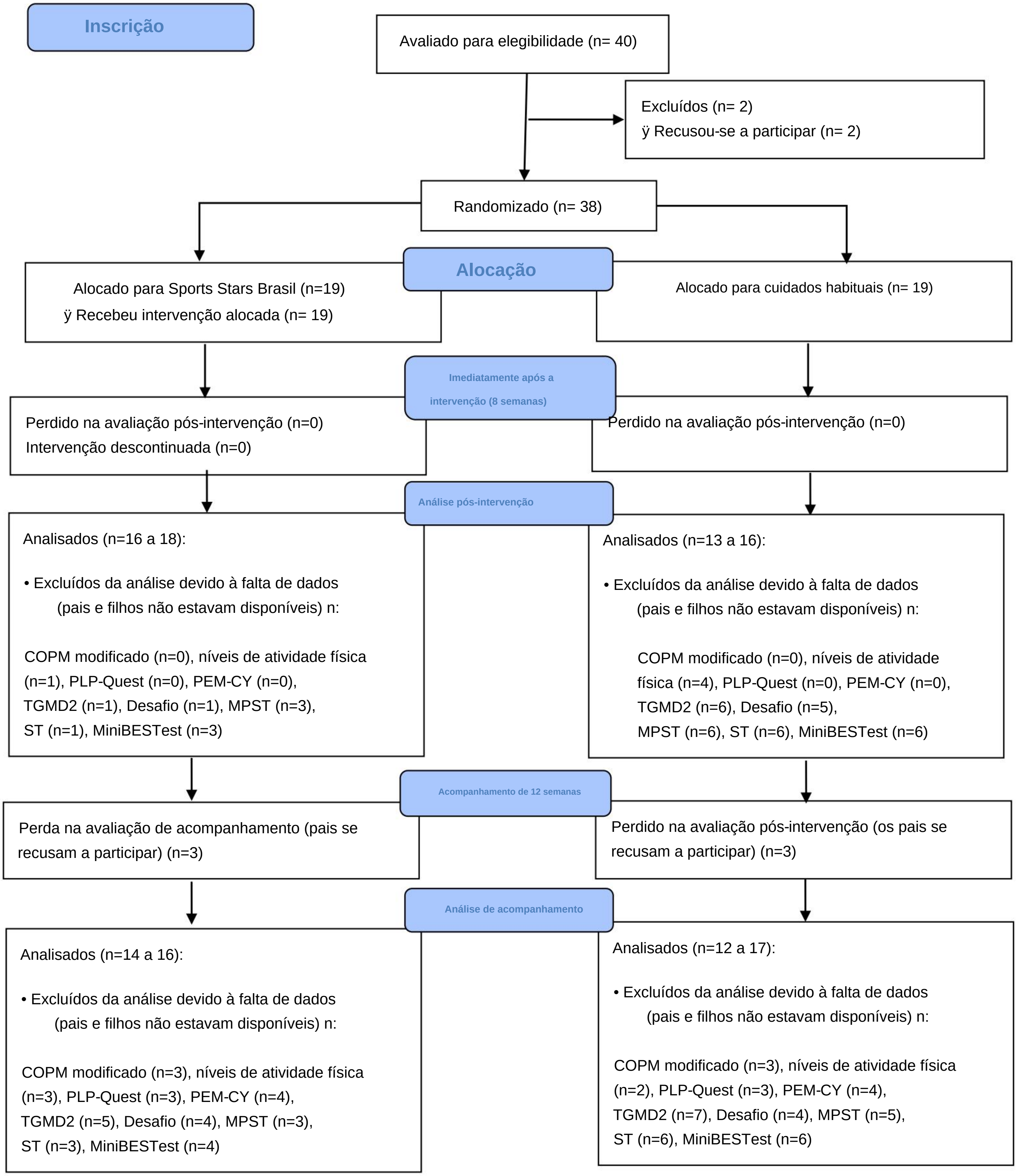

*Legenda: COPM: Medida Canadense de Desempenho Ocupacional; MiniBESTest: Teste de Mini Sistemas de Avaliação de Equilíbrio; MPST: Potência Muscular Teste de Velocidade; PEM-CY: Medida de Participação e Ambiente para Crianças e Jovens; PLP-Quest: Questionário de Perfil de Alfabetização Física; ST: Teste de Velocidade 10x5; TGMD-2: Teste de Desenvolvimento Motor Grosso-2
