# Supplementary material for: Intervenção de esportes modificados para melhorar metas de participação e competências de atividade em crianças deambuladoras com paralisia cerebral: um ensaio clínico randomizado
Source: Dev Med Child Neurol. 2025 Jul 3;68(1):e1–e15. doi: 10.1111/dmcn.16411 (PMC12683299; doi:10.1111/dmcn.16411)
Supplement: Supplementary file 2 — Table S1: Usual therapy group: Physical therapy information according to parents and caregivers. [file DMCN-68-e1-s001.pdf]

Tabela S1. Grupo de terapia usual: Informações sobre fisioterapia segundo pais e cuidadores

Identificação da criança	**Onde?**	**Quanto?**	**Quanto tempo?**	**Contexto**	**Exercícios e intervenções (de acordo com pais e responsáveis)**	**Participaram da maioria das sessões (>50%) ao longo das 8 semanas?**
**1**	Domiciliar	2	45 minutos	Ambiente individual/clínico	Exercícios de equilíbrio, treinamento de força	Sim
**2**	Serviço público	1	45 minutos	Ambiente individual/clínico	Treinamento de força, Treinamento de atividades motoras brutas	Sim
**3**	Serviço público	1	45 minutos	Ambiente individual/clínico	Não relatado	Sim
**4**	Serviço público	1	45 minutos	Ambiente individual/clínico	Não relatado	Não (dificuldades da família em comparecer às seções)
**5**	Serviço público	1	45 minutos	Ambiente individual/clínico	Não relatado	Não
**6**	Serviço privado	1	60 minutos	Ambiente individual/clínico	Treinamento de corrida Exercícios de coordenação e equilíbrio dinâmico	Sim
**7**	Serviço público	1	45 minutos	Ambiente individual/clínico	Não relatado	Não (indisponibilidade de serviços)
**8**	Serviço privado	2	45 minutos	Ambiente individual/clínico	Treinamento em esteira com suporte de peso corporal, treinamento de força	Sim
**9**	Serviço público	2	30 minutos	Ambiente individual/clínico	Exercícios de mobilidade e treinamento de força, treino de corrida na esteira	Sim
**10**	Serviço público	2	45 minutos	Ambiente individual/clínico	Não relatado	Sim
**11**	Serviço público	1	45 minutos	Ambiente individual/clínico	Treinamento de força	Sim
**12**	Serviço público	2	45 minutos	Ambiente individual/clínico	Exercícios de mobilidade articular, treinamento de força, treinamento motor grosso	Sim
**13**	Serviço público	2	45 minutos	Ambiente individual/clínico	Exercícios de mobilidade articular, treinamento de atividades motoras brutas	Sim
**14**	Serviço público	2	45 minutos	Ambiente individual/clínico	Exercícios de equilíbrio, treinamento em esteira Treinamento de atividades motoras brutas	Sim

**15**	Serviço público	3	45 minutos	Ambiente individual/clínico	Não relatado	Sim
**16**	Serviço privado	1	45 minutos	Ambiente individual/clínico	Não relatado	Sim
**17**	Serviço público	1	45 minutos	Ambiente individual/clínico	Treinamento de força, circuito	Sim
**18**	Serviço público	2	45 minutos	Ambiente individual/clínico	Treinamento de atividades motoras brutas	Sim
**19**	Serviço privado	2	60 minutos	Ambiente individual/clínico	Treinamento de atividades motoras brutas, terapia de jogos, exercícios de equilíbrio	Sim
