# Supplementary material for: Intervenção de esportes modificados para melhorar metas de participação e competências de atividade em crianças deambuladoras com paralisia cerebral: um ensaio clínico randomizado
Source: Dev Med Child Neurol. 2025 Jul 3;68(1):e1–e15. doi: 10.1111/dmcn.16411 (PMC12683299; doi:10.1111/dmcn.16411)
Supplement: Supplementary file 3 — Table S2: Changes over the time for Sports Stars Brasil and usual physical therapy groups. [file DMCN-68-e1-s002.pdf]

Tabela S2. Mudanças ao longo do tempo para os grupos Sports Stars Brasil e Fisioterapia Usual

Resultados primários														
	Sports Stars Brasil							Fisioterapia usual						
	Linha de base Média (DP)	Pós-intervenção Média (DP)	Follow-up Média (DP)	Diferença pós-intervenção	*d*	Diferença no follow-up*	*d*	Linha de base Média (DP)	Pós-intervenção Média (DP)	Follow-up Média (DP)	Diferença pós-intervenção*	*d*	Diferença de acompanhamento*	*d*
**Meta de participação e envolvimento - Desempenho Medido pelo COPM**	4,94 (1,80)	7,15 (2,33)	7,25 (2,48)	2.21	1,72	2.31	1,06	5,31 (1,85)	5,68 (2,18)	6,75 (1,73)	0,37	0,18	1,44	0,80
**Participação envolvimento meta-Satisfação Medido pelo COPM**	4,94 (2,50)	7,73 (2,28)	8,00 (2,25)	2,79	1,99	3.06	1.18	4,52 (2,29)	5,21 (2,67)	6,43 (2,36)	0,69	0,27	1,91	0,82
**Meta de participação e frequência - Desempenho Medido pelo COPM**	3,00 (2,47)	5,77 (3,42)	6,33 (3,43)	2,77	0,30	3,33	1.11	2,33 (1,97)	3,05 (2,53)	3,73 (2,98)	0,72	0,31	1,40	0,55
**Meta de participação e frequência - Satisfação Medido pelo COPM**	2,55 (2,40)	6,16 (3,71)	6,60 (3,52)	3,00	0,48	3,44	1,34	3,16 (3,05)	3,50 (3,24)	4,26 (3,59)	0,95	0,36	1,71	0,33
**Resultados secundários**														
	Sports Stars Brasil							Fisioterapia usual						
	Linha de base Média (DP)	Pós-intervenção Média (DP)	Follow-up Média (DP)	Diferença pós-intervenção	*d*	Diferença no follow-up*	*d*	Linha de base Média (DP)	Pós-intervenção Média (DP)	Follow-up Média (DP)	Diferença pós-intervenção*	*d*	Diferença de acompanhamento*	*d*
**Objetivo de desempenho motor - Desempenho Medido pelo COPM**	4,57 (1,74)	7,21 (1,96)	7,71 (1,82)	2,64	1,42	3.14	0,64	5,26 1,66)	5,68 (1,85)	6,06 (1,87)	0,42	0,23	1,49	0,47

**Meta de desempenho motor-Satisfação Medido pelo COPM**	5,47 (2,16)	8.31(2.10)	8,56 (1,89)	2,84	0,84	3.09	1,15	5,68 (2,23)	6,05 (2,23)	6,50(2,65)	0,37	0,16	0,82	0,33
**Níveis de atividade física - Comportamento sedentário Medido pelo acelerômetro**	59,45 (13,73)	57,29 (11,86)	55,52 (10,97)	-2,16	0,16	-3,93	0,31	54,48 (9,94)	57,01 (8,60)	55,73 (10,45)	1,25	0,10	2,53	0,12
	**Estrelas do Esporte Brasil**							**Cuidados de Fisioterapia Habituais**						
	**Linha de base Média (DP)**	**Pós-intervenção Média (DP)**	**Seguir Média (DP)**	**Diferença pós-intervenção**	***d***	**Diferença de acompanhamento***	***d***	**Linha de base Média (DP)**	**Pós-intervenção Média (DP)**	**Seguir Média (DP)**	**Diferença pós-intervenção***	***d***	**Diferença de acompanhamento***	***d***
**Níveis de atividade física - Tempo em LPA Medido pelo acelerômetro**	30.11 (10.01)	31,52 (8,09)	32,19 (7,72)	1,41	0,15	2.18	0,23	33,62 (5,70)	32,07 (5,06)	33,34 (8,06)	-1,55	0,28	-0,28	0,04
**Níveis de atividade física - Tempo em AFMV Medido pelo acelerômetro**	10,37 (4,35)	11,17 (4,58)	12,18 (4,78)	0,80	0,17	1,81	0,39	11,87 (4,88)	10,86 (4,11)	10,53 (3,46)	-0,70	0,22	-1,34	0,31
**Níveis de atividade física - AFMV média/semana Medido pelo acelerômetro**	65,63(28,68)	64,29 (32,81)	68,56 (31,32)	-1,34	0,04	2,93	1,09	69,05(20,64)	61,53 (21,89)	51,76 (20,52)	-7,52	0,35	-17,29	0,84
**Alfabetização física Medido pelo PLP-Quest**	69,94 (15,79)	81,69 (15,02)	79,05 (19,12)	11,75	1,08	9.11	0,53	70,45 (17,29)	75,43 (15,98)	72,05 (20,99)	4,98	0,29	1,60	0,08
**Capacidade motora e locomotora Medido pelo TGMD2**	29,15 (10,34)	32,33 (9,57)	34,66 (9,16)	3.18	0,31	5,51	0,56	31,22 (9,34)	37,38 (9,81)	32,16 (9,90)	6.16	0,64	0,94	0,09

**Capacidade de controle de objetos de habilidades motoras Medido pelo TGMD2**	28,40 (10,67)	34,55 (7,42)	38,00 (5,73)	6.15	0,66	9,60	1.12	31,23 (8,73)	33,23 (6,98)	33,46 (8,15)	2,00	0,25	2.23	0,27
**Capacidade geral de habilidades motoras Medido pelo TGMD2**	57,55 (18,72)	66,88 (15,86)	72,92 (13,68)	9,33	0,75	15,37	0,93	62,47 (17,15)	70,61 (16,00)	65,08 (16,53)	7,84	0,49	2,61	0,17
**Capacidade de habilidades motoras Medido pelo teste de desafio**	35,84 (20,10)	41,40 (21,57)	42,72 (19,10)	5,56	0,26	6,88	0,35	40,55 (21,23)	47,57 (24,67)	42,43 (24,23)	7.02	0,10	1,88	0,08
**Equilíbrio Medido pelo Kids Mini BESTest**	26,25 (4,20)	25,56 (6,98)	26,73 (3,57)	-0,69	0,11	0,48	0,12	27,52 (2,83)	28,38 (2,46)	27,23 (3,39)	0,86	0,32	-0,29	0,09
**Potência Muscular - Média Medido pelo MPST**	57,55 (18,72)	75,23 (41,96)	73,41 (40,56)	-6,21	0,14	4,39	0,09	62,47 (17,15)	79,19 (66,57)	56,16 (42,43)	1,76	0,24	-21,24	0,01
**Potência Muscular - Pico Medido pelo MPST**	81,55 (47,61)	93,33 (51,68)	91,36 (44,62)	11,78	0,52	9,81	0,18	95,32 (64,17)	94,09 (72,84)	79,72 (59,98)	-1,23	0,35	-15,60	0,25
**Agilidade Medido por 10X5 ST**		39,16 (19,79)	35,37 (6,26)	2,67	0,16	-1,12	0,11	36,03 (11,95)	35,26 (8,68)	37,20 (7,95)	-0,77	0,07	1.17	0,11
	**Estrelas do Esporte Brasil**							**Cuidados de Fisioterapia Habituais**						
	**Linha de base Média (DP)**	**Pós-intervenção Média (DP)**	**Seguir Média (DP)**	**Diferença pós-intervenção**	***d***	**Diferença de acompanhamento***	***d***	**Linha de base Média (DP)**	**Pós-intervenção Média (DP)**	**Seguir Média (DP)**	**Diferença pós-intervenção***	***d***	**Diferença de acompanhamento***	***d***
**Participação geral - Frequência escolar Medido por PEM-CY**	5,36 (1,53)	5,26 (1,28)	5,33 (1,23)	-0,10	0,09	-0,03	0,02	5,15 (1,80)	4,84 (1,64)	5,26 (1,38)	-0,31	0,18	0,11	0,06

**Participação geral - Envolvimento escolar Medido por PEM-CY**	4,42 (0,76)	4,47 (0,61)	4,60 (0,73)	0,05	0,63	0,18	0,24	3,89 (1,24)	4,15 (1,21)	4,33 (0,89)	0,26	0,21	0,44	0,40
**Participação geral - Desejo de mudança da escola Medido por PEM-CY**	64,21 (34,36)	55,78 (35,63)	54,66 (34,19)	-8,43	0,24	-9,55	0,27	71,57 (37,30)	63,15 (34,80)	54,66 (26,69)	-16,91	0,23	-16,91	0,52
**Participação geral - Número de atividades da escola Medido por PEM-CY**	3,00 (1,41)	3,94 (1,12)	3,60 (1,24)	0,37	0,73	0,03	0,45	3,57 (1,12)	3,52 (1,42)	3,60 (1,40)	0,52	0,03	0,60	0,02
**Participação geral - Frequência da comunidade Medido por PEM-CY**	4,42 (1,26)	4,47 (1,34)	4,60 (1,12)	0,05	0,03	0,18	0,32	4,42 (1,16)	4,26 (1,09)	4,40 (1,05)	-0,16	0,14	-0,02	0,01
**Participação geral - Envolvimento da comunidade Medido por PEM-CY**	4,31 (0,67)	4,52 (0,77)	4,60 (0,63)	0,21	0,29	0,29	0,29	4,47 (0,69)	4,63 (0,68)	4,60 (0,63)	0,16	0,23	0,13	0,19
**Participação geral - Desejo da comunidade por mudança medido pelo PEM-CY**	49,33 (28,65)	64,21(25,45)	68,84(24,87)	14,88	0,59	19,51	0,72	44,66(28,75)	56,31 (30,40)	62,63 (29,97)	11,65	0,39	17,97	0,61
**Participação geral - Número de atividades da comunidade Medido por PEM-CY**	5,52 (2,31)	6,15 (2,00)	5,86 (2,26)	0,63	0,29	0,34	0,14	5,57 (2,09)	5,94 (2,06)	6,46 (2,16)	0,37	0,17	0,89	0,41

Legenda: 10X5ST- Teste de Velocidade de 10 metros por 5 segundos, CI- Intervalos de confiança COPM- Medida Canadense de Desempenho Ocupacional; LPA- Atividade Física Leve, AFMV- Atividade Física Moderada a Vigorosa; MPST- Teste de Velocidade de Potência Muscular, PEM-CY- Medida de Participação e Ambiente para Crianças e Jovens; TGMD-2- Teste de Desenvolvimento Motor Bruto Segunda Edição; DP- Desvio Padrão
